# Conjugated Hyperbilirubinemia in Early Infancy: Rethinking Diagnostic Cut-Offs—A Retrospective Analysis

**DOI:** 10.3390/ijns12020033

**Published:** 2026-05-11

**Authors:** Daniel Pfurtscheller, Carola Ganzer, Ena Suppan, Melina Winkler, Bernhard Schwaberger, Lisa Sallmon, Gerhard Pichler, Benno Kohlmaier

**Affiliations:** 1 Division of Neonatology, Department of Pediatrics and Adolescent Medicine, Medical University of Graz, 8036 Graz, Austria; daniel.pfurtscheller@medunigraz.at (D.P.); carola.ganzer@stud.medunigraz.at (C.G.); ena.suppan@medunigraz.at (E.S.); melina.winkler@medunigraz.at (M.W.); bernhard.schwaberger@medunigraz.at (B.S.); lisa.sallmon@medunigraz.at (L.S.); gerhard.pichler@medunigraz.at (G.P.); 2 Division of General Pediatrics, Department of Pediatrics and Adolescent Medicine, Medical University of Graz, 8036 Graz, Austria

**Keywords:** conjugated hyperbilirubinemia, neonatal cholestatic liver disease, biliary atresia, bilirubin cut-off values

## Abstract

Background: Conjugated hyperbilirubinemia in early infancy is a critical indicator of hepatobiliary dysfunction. Prompt and accurate identification is essential to diagnose cholestatic liver disease (CLD), particularly biliary atresia. Current guidelines define conjugated bilirubin (CB) ≥ 1 mg/dL as abnormal, irrespective of total bilirubin (TB). This study aimed to evaluate whether combining absolute and relative CB thresholds improves diagnostic performance for CLD. Methods: We retrospectively analyzed all infants aged ≤6 months of chronological age with CB ≥ 1 mg/dL admitted to the Department of Pediatrics and Adolescent Medicine, Medical University of Graz, Austria, between January 2004 and February 2025. During that period, 116,104 infants were born at our hospital catchment area; 3119 of these underwent bilirubin fractionation, and 257 infants (0.2% of total births) had a CB ≥ 1 mg/dL and were included in the analysis. Clinical and biochemical data were extracted. Diagnostic performance of the absolute (CB ≥ 1 mg/dL) and in combination with the relative (CB ≥ 20% of TB) thresholds was assessed using receiver operating characteristic (ROC) analysis for the detection of CLD. Results: Among 257 infants, 47 (18%) were diagnosed with CLD. The median age at the time of blood sampling was 18 days (IQR 9–31). The combined criterion (CB ≥ 1 mg/dL and ≥20% of TB) achieved 100% sensitivity and 61.2% specificity (AUC = 0.82, 95% CI 0.79–0.92; *p* < 0.001). Implementation of the combined cut-off reduced the number needed to screen from 5.5 to 2.7, representing nearly a twofold improvement in diagnostic efficiency. Conclusions: Applying both absolute (≥1 mg/dL) and relative (≥20% of total bilirubin) CB thresholds substantially improves detection of neonatal CLD in early infancy. This combined approach maintains full sensitivity while reducing false positives and unnecessary investigations, thereby enhancing diagnostic efficiency in early infancy.

## 1. Introduction

Neonatal jaundice is among the most common clinical findings in early infancy and is characterized by elevated bilirubin concentrations in the blood. In most cases, it reflects a transient physiological adaptation to extrauterine life and resolves spontaneously without the need for treatment. However, jaundice may also represent the first sign of significant hepatobiliary disease, such as biliary atresia or neonatal hepatitis, conditions in which timely detection is crucial [1,2]. Distinguishing physiological from pathological jaundice in early infancy is challenging, as visual assessment alone cannot reliably differentiate conjugated from unconjugated hyperbilirubinemia [1].

Bilirubin is generated through the normal degradation of heme following erythrocyte breakdown. Heme is first converted to biliverdin by heme oxygenase and subsequently reduced to bilirubin by biliverdin reductase [3]. In its unconjugated form, bilirubin is lipophilic and circulates bound to albumin until hepatic uptake. Within hepatocytes, conjugation by uridine diphosphate-glucuronosyltransferase (UGT1A1) converts bilirubin into a water-soluble form, enabling excretion into bile and elimination through the intestines [4,5]. Disruption of this pathway, especially impaired bile flow, causes conjugated hyperbilirubinemia and clinically manifests as cholestatic jaundice.

While mild unconjugated hyperbilirubinemia is typically benign and self-limiting, elevated conjugated bilirubin (CB) concentrations always warrant further evaluation, as they reflect impaired bile formation or excretion due to hepatocellular injury or biliary obstruction [6]. Early recognition of conjugated hyperbilirubinemia is essential because delayed diagnosis of cholestatic liver disease (CLD), especially biliary atresia, can severely affect long-term outcomes and native liver survival [7].

According to current international guidelines from European Society for Pediatric Gastroenterology, Hepatology and Nutrition (ESPGHAN) and North American Society for Pediatric Gastroenterology, Hepatology, and Nutrition (NASPGHAN), a CB value of ≥1 mg/dL is considered pathological, regardless of the total bilirubin (TB) concentration [7]. Although this absolute cut-off is highly sensitive, it may result in a considerable number of unnecessary investigations, particularly in early neonatal period when bilirubin metabolism and bile flow are still maturing. Several studies have shown that healthy infants may transiently exceed this limit without any underlying CLD [2,5,8].

Rather than relying solely on an absolute cut-off, the proportion of CB relative to TB has been suggested as an alternative diagnostic indicator [8,9,10,11]. Such a ratio accounts for physiological variability in bilirubin metabolism and may improve diagnostic specificity while maintaining high sensitivity for detecting clinically relevant cholestasis. However, consensus regarding the optimal threshold is lacking, and available evidence is limited by small sample sizes or heterogeneous study designs.

This retrospective study aimed to evaluate whether a conjugated bilirubin fraction ≥ 20% of TB improves the specificity for diagnosing CLD in infants with CB ≥ 1 mg/dL without loss of sensitivity.

## 2. Materials and Methods

### 2.1. Study Design

This retrospective observational study was conducted at the Department of Pediatrics and Adolescent Medicine, Medical University of Graz, Austria. Ethical approval was obtained from the institutional Ethics Committee of the Medical University of Graz (EK: 1002/2025). The study was conducted in accordance with the Declaration of Helsinki and complied with institutional data protection regulations.

Inclusion and Exclusion Criteria

All infants aged ≤6 months of chronological age who were admitted to the University Hospital Graz between January 2004 and February 2025 and had a CB concentration of ≥1 mg/dL were eligible for inclusion.

According to the ESPGHAN guidelines [7], infants with CB < 1 mg/dL were not included, as clinically relevant CLD is considered unlikely in neonates with CB levels below this threshold.

The only exclusion criterion was an age > 6 months at the time of the relevant blood sampling. No additional exclusion criteria were applied.

Hospital setting

This study was performed at a tertiary-level university hospital serving a catchment area of approximately 450,000 inhabitants. The hospital oversees five maternity wards with an average of 6000–7000 live births annually.

### 2.2. Data Collection

Clinical and laboratory data were retrospectively extracted from the electronic medical records (Medocs^®^ system).

The following parameters were retrieved.

- Demographic data: age (in days) at the time of blood sampling.
- Laboratory parameters: total, conjugated, and unconjugated bilirubin; alkaline phosphatase (AP); gamma-glutamyltransferase (GGT); cholinesterase (CHE); aspartate aminotransferase (AST); alanine aminotransferase (ALT); creatine kinase (CK); and lactate dehydrogenase (LDH).
- Clinical data: presence or absence of a CLD diagnosis and whether an abdominal ultrasonography of the hepatobiliary system was performed.

The proportion of CB relative to TB was calculated. In addition, due to access to a comprehensive regional electronic health record system, all hospital-based medical encounters within the federal state could be reviewed longitudinally. This enabled complete follow-up of all patients, including those who did not undergo ultrasonography, ensuring that subsequent diagnoses of hepatobiliary disease would have been captured.

All data were pseudonymized before analysis to ensure compliance with data protection regulations.

### 2.3. Statistical Analysis

Analyses were performed using IBM SPSS Statistics, Version 29.0 (IBM Corp., Armonk, NY, USA). Descriptive statistics were used to summarize all variables. The Shapiro–Wilk test was applied to assess normality. Normally distributed variables were expressed as mean ± standard deviation (SD); whereas non-normally distributed variables were summarized as median and interquartile range (IQR).

To compare groups of infants with and without documented liver disease, the Mann–Whitney U test was used for non-parametric variables. To explore potential confounding by prematurity and parenteral nutrition exposure, we additionally performed stratified subgroup analyses for preterm versus term infants and for infants with versus without parenteral nutrition. Statistical significance was defined as a two-sided *p*-value < 0.05.

To evaluate the diagnostic performance of the relative CB fraction, a receiver operating characteristic (ROC) analysis was performed. The test variable was the percentage of conjugated bilirubin, and the target variable was the presence of liver disease. The area under the curve (AUC) was calculated to assess discriminative ability, and the Youden Index (J = sensitivity + specificity − 1) was used to determine the optimal cut-off point.

Sensitivity, specificity, and number needed to screen (NNS) were determined manually. Diagnostic performance was compared between the established absolute CB threshold (≥1 mg/dL) and the combined strategy incorporating an additional relative cut-off (CB ≥ 20% of TB). All reported percentages and derived measures are rounded to one decimal place.

## 3. Results

### 3.1. Study Population

**Figure 1:**
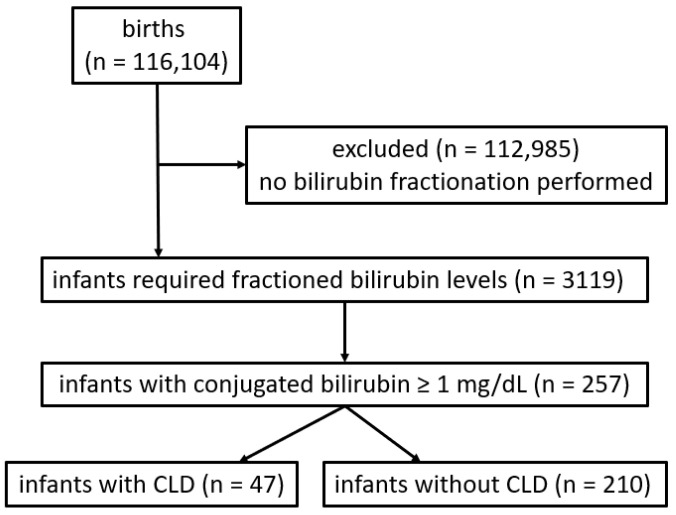
Inclusion and exclusion of infants with and without cholestatic liver disease (CLD).

During the study period, a total of 116,104 infants were born within the hospital’s catchment area. Of these, 3119 infants (2.7%) underwent bilirubin fractionation, and 257 infants had a CB concentration ≥ 1 mg/dL and were therefore included in the analysis (Figure 1).

The median age at the time of blood sampling was 18 days (IQR 9–31). Of these, 126 (49%) were male and 131 (51%) were female. Among these 257 infants, 47 (18%) were diagnosed with a CLD whereas 210 (82%) had no evidence of hepatic pathology. An abdominal ultrasonography was performed in only 198 (77%) patients; however, longitudinal follow-up data were available for all patients of the total cohort.

The cohort consisted of 189 term-born (74%) and 68 preterm (26%) infants, with a median gestational age of 39 + 5 weeks (range: 25 + 1–41 + 6).

Among the 47 infants diagnosed with CLD, biliary atresia was the most frequent single diagnosis, accounting for 14 cases (30%). Alpha-1 antitrypsin deficiency was identified in three infants (6%). In another three infants (6%), a genetic disorder—such as progressive familial intrahepatic cholestasis—was identified as the underlying cause of cholestasis. Additionally, Alagille syndrome and an inborn error of metabolism were each diagnosed in two infants (4%); these included one case of Wolman syndrome and one case of galactosemia.

**Figure 2:**
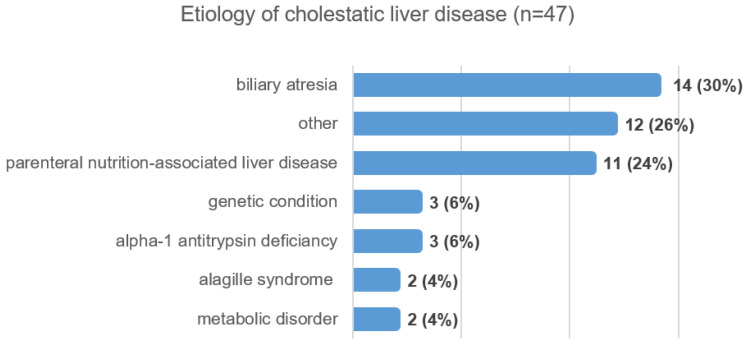
Infants diagnosed with cholestatic liver disease.

Eleven infants (24%) required prolonged parenteral nutrition due to various gastrointestinal diseases and received intensive care. In these cases, a reversible cholestasis was observed, characterized by elevated liver biochemistry and corresponding sonographic findings, primarily attributable to parenteral nutrition-associated cholestasis. Twelve cases (26%) were classified as “other,” comprising a heterogeneous group of diagnoses, including hepatotropic viral infections, alloimmune reactions, liver hematomas, and mechanical obstruction of bile flow (Figure 2).

### 3.2. Laboratory Findings

The overall median TB concentration was 9.8 mg/dL (IQR 5.1–14.2), and the median CB was 1.5 mg/dL (IQR 1.2–2.3). The median proportion of CB relative to TB across the entire cohort was 18.8% (IQR 10.3–47.9).

Infants diagnosed with CLD showed significantly higher CB levels compared with infants without CLD. The relative CB fraction was also significantly increased in the CLD group in comparison to the non-CLD group.

Liver-associated enzymes (AP, AST, ALT, GGT) were significantly higher in infants with CLD, whereas CHE and CK did not differ significantly between groups (Table 1).

### 3.3. Diagnostic Performance

ROC analysis demonstrated good diagnostic discrimination of the relative CB fraction for identifying cholestatic liver disease (AUC = 0.82, 95% CI 0.79–0.92; *p* < 0.001).

The optimal cut-off determined by the Youden Index was a CB fraction ≥ 20% of TB. Using this threshold, sensitivity was 100% and specificity increased to 61.2%, yielding a positive predictive value of 37.2%.

Importantly, the conventional absolute threshold of CB ≥ 1 mg/dL served as the inclusion criterion for this cohort. Thus, its sensitivity was inherently 100% by design, while specificity could not be determined (i.e., effectively 0%), as no infants with CB < 1 mg/dL were included for comparison. Consequently, this threshold resulted in a high number of false-positive classifications when used as a screening test.

Adding the relative threshold (CB ≥ 20% of TB) to the absolute criterion (CB ≥ 1 mg/dL) substantially improved diagnostic performance by reducing false positives without missing any cases of CLD. Accordingly, the number needed to screen (NNS) improved from 5.5 using the absolute criterion alone to 2.7 with the combined approach (Table 2), representing almost a twofold increase in screening efficiency. In additional stratified analyses, the combined criterion maintained 100% sensitivity across clinically relevant subgroups. Specifically, all infants with CLD were identified both among those with parenteral nutrition exposure (11/11) and those without parenteral nutrition exposure (36/36). Likewise, sensitivity remained 100% in both preterm infants (13/13) and term infants (34/34).

## 4. Discussion

This retrospective study evaluated whether applying a relative cut-off of ≥20% CB of TB improves the specificity for diagnosing CLD in infants with CB ≥ 1 mg/dL, without loss of sensitivity. Our findings demonstrate that combining the established absolute CB threshold with a relative CB fraction ≥ 20% enhances diagnostic accuracy compared with the absolute cut-off alone. Importantly, this approach substantially reduced unnecessary follow-up investigations while maintaining complete sensitivity for clinically relevant cholestatic liver disease.

Current ESPGHAN and NASPGHAN guidelines recommend further diagnostic evaluation for all infants with a CB concentration ≥ 1 mg/dL, irrespective of TB levels [7]. This absolute cut-off was designed to maximize sensitivity and ensure early recognition of severe conditions such as biliary atresia. However, this approach may generate a high number of false positives, as even healthy neonates can transiently exhibit CB levels near or slightly above this threshold during the early neonatal period [2,5,7,12].

Physiologically, bilirubin metabolism in neonates is a dynamic and developmentally regulated process. Following birth, the hepatocellular conjugation capacity gradually increases due to rising activity of UGT1A1, while bile flow, enterohepatic circulation, and intestinal microbiota remain immature [3,5,7]. In this phase, both TB and CB levels fluctuate markedly, often without pathological significance. Consequently, reliance on a single absolute bilirubin cut-off may inadequately reflect the physiological transition of the hepatobiliary system in early life.

Moreover, the literature remains inconsistent regarding what constitutes “pathological” conjugated hyperbilirubinemia. Reported absolute CB thresholds vary from 0.8 mg/dL to 2 mg/dL, depending on study population, laboratory methodology, and clinical context [8,9,10,11]. Some authors have therefore proposed relative measures—such as the proportion of CB relative to TB—as a more physiologically meaningful indicator of impaired bile formation or excretion [8]. In routine practice, clinical assessment or transcutaneous bilirubin measurements provide only a rough estimate of jaundice severity and cannot distinguish between unconjugated and conjugated bilirubin fractions, reinforcing the necessity of quantitative laboratory evaluation [1,2].

While the absolute CB threshold of ≥1 mg/dL ensured complete sensitivity within our cohort, its discriminatory value was limited by the large number of false-positive classifications. In contrast, applying an additional relative CB cut-off of ≥20% substantially increased specificity while preserving full sensitivity.

The optimal cut-off derived from ROC analysis was approximately 20%, directly supporting the use of this threshold in clinical practice. While no universally accepted relative threshold exists, prior studies have highlighted the importance of interpreting conjugated bilirubin in relation to total bilirubin rather than relying solely on absolute values [8,11]. From a physiological standpoint, higher relative proportions of conjugated bilirubin likely reflect impaired biliary excretion rather than normal neonatal adaptation.

Our findings support this concept. According to current guidelines [7], any infant with CB ≥ 1 mg/dL should undergo further evaluation for cholestasis to correctly identify almost all infants with CLD. However, this approach, resulting in many false-positive referrals when applied alone and therefore led to many unnecessary diagnostic procedures in healthy infants.

Our study showed that adding a relative cut-off of CB ≥ 20% of TB reduced false-positive classifications within this at-risk cohort of infants already exceeding the absolute threshold, while maintaining full sensitivity. Importantly, this finding remained unchanged in stratified subgroup analyses according to prematurity and parenteral nutrition exposure, suggesting that the diagnostic performance of the combined threshold was not driven solely by these clinically relevant subgroups. Thus, the combined criterion improves the triage value of CB screening without risking missed cases of clinically relevant cholestasis.

These results align with previous studies demonstrating greater diagnostic precision using relative bilirubin measures [13,14,15] and with the pathophysiological framework proposed by Feldman and Sokol [8], which emphasizes the balance between bilirubin conjugation and excretion rather than absolute concentrations alone [8,9,16,17]. Recent evidence from a 2024 systematic review and meta-analysis further supports the use of direct/conjugated bilirubin as a highly accurate screening tool for biliary atresia, particularly in the early neonatal period [18]. Importantly, this analysis also highlighted the potential benefit of combining screening approaches to optimize diagnostic performance. In this context, our findings extend current evidence by demonstrating that the addition of a relative bilirubin threshold (≥20%) can substantially improve specificity within the population identified by absolute screening criteria, without compromising sensitivity. This suggests that bilirubin-based screening strategies may benefit from a two-step approach, combining high sensitivity in initial detection with improved specificity in subsequent risk stratification.

From a clinical perspective, incorporating a relative CB threshold accounts for interindividual variability and developmental physiology. This approach enables a more nuanced interpretation of laboratory results and may help prevent overdiagnosis. In our cohort, nearly two thirds of follow-up imaging and laboratory investigations could have been avoided by additionally applying the combined criterion, without missing any cases of clinically relevant CLD. Beyond reducing unnecessary diagnostic procedures, this approach may also alleviate parental anxiety, decrease overall healthcare costs, and optimize allocation of clinical resources.

### 4.1. Clinical Implications

The early distinction between benign and pathological jaundice remains a critical challenge in neonatal care. Delayed diagnosis of cholestasis, particularly biliary atresia, can have severe consequences. Timely surgical intervention, such as Kasai portoenterostomy performed within 60 days of life, significantly improves native liver survival and long-term outcomes [19]. At the same time, excessive investigation of healthy infants imposes avoidable stress on families and strains healthcare systems.

Incorporating a relative CB threshold into standard evaluation could help balance these competing priorities. Automated calculation and reporting of the CB-to-TB ratio in patients with CB ≥ 1 mg/dL could easily be implemented within existing electronic laboratory systems. Flagging relative CB fractions ≥ 20% may facilitate earlier recognition of clinically relevant cholestasis, particularly in centers without specialized pediatric hepatology expertise, where delayed diagnosis of biliary atresia and other cholestatic conditions remain a concern [2,4,5].

### 4.2. Strengths and Limitations

The main strengths of this study include the relatively large sample size and the consistent laboratory methodology. The uniform setting of a single tertiary care hospital further enhances internal consistency and minimizes methodological variability.

However, several limitations must be acknowledged. The retrospective, single-center design limits generalizability, and selection bias cannot be excluded, as only infants who underwent bilirubin fractionation were included. Incomplete follow-up may have resulted in an underestimation of delayed or mild cases of CLD although this is unlikely to have influenced the overall conclusions. Potential confounders such as prematurity, hemolysis, infection, and parenteral nutrition exposure were not assessed in a formal multivariable model. Nevertheless, stratified subgroup analyses demonstrated that the combined threshold retained 100% sensitivity in both preterm and term infants as well as in infants with and without parenteral nutrition exposure, supporting the robustness of the observed diagnostic performance. A further limitation of our study is that we did not include infants with CB concentrations < 1 mg/dL. While this precludes assessment of diagnostic performance below the guideline-recommended threshold, current international guidelines (e.g., ESPGHAN and NASPGHAN) do not recommend routine hepatobiliary evaluation in this group, and such cases are therefore not systematically captured in clinical practice. Previous large-scale studies have demonstrated that this threshold is associated with excellent discriminatory ability for identifying neonates at risk, with exceptionally high sensitivity. Restricting the cohort to infants meeting this threshold therefore ensured alignment with current clinical practice and avoided dilution of diagnostic performance estimates [7]. Furthermore, the long study period (2004–2025) may introduce temporal bias, as laboratory techniques and clinical management strategies have evolved over time. Although all analyses were performed within a single center using standardized methods, residual effects of temporal variation cannot be fully excluded.

### 4.3. Future Perspectives

Future research should aim to validate the combination of an absolute CB threshold of ≥1 mg/dL and a relative cut-off of 20% in prospective, multicenter cohorts and to evaluate whether this approach can be integrated into standardized neonatal screening algorithms [9,16,17,18]. Such studies should also evaluate performance across different clinical settings and laboratory platforms.

Additionally, cost-effectiveness analyses should be conducted to assess the potential healthcare savings associated with reducing unnecessary diagnostic testing, imaging, and hospitalizations. Finally, integrating automated bilirubin ratio calculations into electronic health record systems could facilitate standardized implementation and enable timely identification of high-risk infants for CLD across different levels of care [15].

## 5. Conclusions

In summary, this study demonstrates that adding the relative proportion of CB to TB to the absolute CB value provides a physiologically sound and clinically valuable parameter for identifying neonatal cholestasis. A relative threshold of approximately 20% preserves the sensitivity of the conventional absolute criterion while adding clinically relevant specificity, thereby reducing unnecessary diagnostic workups without missing clinically significant disease. Incorporating this simple metric into standard bilirubin assessment could meaningfully refine current neonatal screening practices and facilitate earlier diagnosis and improved outcomes in infants with CLD.

## Figures and Tables

**Table 1:** Infants with and without a cholestatic liver disease (CLD). Data for the following are presented: alkaline phosphatase (AP), alanine aminotransferase (ALT), aspartate aminotransferase (AST), cholinesterase (CHE), creatine kinase (CK), gamma-glutamyltransferase (GGT), lactate dehydrogenase (LDH).

	With a Cholestatic Liver Diseases	Without a Cholestatic Liver Diseases	*p*-Value
AP	500 (409–625) U/L	189 (153–291) U/L	<0.001
GGT	347 (289–864) U/L	140 (132–224) U/L	0.012
CHE	4682 (3525–6377) U/L	5285 (3794–6216) U/L	0.206
AST	123 (102–370) U/L	36 (12–250) U/L	0.004
ALT	71 (20–270) U/L	15 (12–64) U/L	0.008
CK	89 (78–186) U/L	141(132–247) U/L	0.178
LDH	328 (255–670) U/L	389 (348–724) U/L	0.263
absolute level of conjugated bilirubin	3.8 (3.2–9.1) mg/dL	1.34 (1.16–1.78) mg/dL	<0.001
relative level of conjugated bilirubin	47.9 (41.2–61.9)%	16.7 (10.7–44.5)%	<0.001

**Table 2:** Comparison of diagnostic thresholds. PPV, positive predictive value; NNS, number needed to screen.

	Sensitivity	Specificity	PPV	NNS
Conjugated bilirubin ≥ 1 mg/dL	1.000	0.000	0.183 (18.3%)	5.5
Conjugated bilirubin ≥ 1 mg/dL + ≥20% of total bilirubin	1.000	0.612 (61.2%)	0.372 (37.2%)	2.7

## Data Availability

The data presented in this study are not publicly available due to privacy and ethical restrictions. De-identified data may be made available by the corresponding author upon reasonable request and with permission from the Ethics Committee of the Medical University of Graz.

## Abbreviations

The following abbreviations are used in this manuscript:

ALT	alanine aminotransferase
AP	alkaline phosphatase
AST	aspartate aminotransferase
AUC	area under the curve
CB	conjugated bilirubin
CHE	cholinesterase
CI	confidence interval
CK	creatine kinase
CLD	cholestatic liver disease
ESPGHAN	European Society for Pediatric Gastroenterology, Hepatology and Nutrition
GGT	gamma-glutamyltransferase
IQR	interquartile range
LDH	lactate dehydrogenase
NASPGHAN	North American Society for Pediatric Gastroenterology, Hepatology, and Nutrition
NNS	number needed to screen
PPV	positive predictive value
ROC	receiver operating characteristic
SD	standard deviation
TB	total bilirubin
UGT1A1	uridine diphosphate-glucuronosyltransferase 1A1
